# Fœtal Auscultation

**Published:** 1860-05

**Authors:** Francis Adams

**Affiliations:** The Editor of the Medical Times and Gazette


### ON FŒTAL ASCULATION.

LETTER FROM DR. FRANCIS ADAMS TO THE EDITOR OF THE MEDICAL TIMES AND GAZETTE.

Sir:—From the statements which have been made under the present head in this journal, it would appear, contrary to my expectation, that there is such a discrepancy in the views of the stethoscopists regarding the sounds detected by asculation of the heart and of the uterus, that I consider it necessary to determine, if possible with precision, what may be assumed as the generally admitted facts of the case, before attempting to settle those points about which a difference of opinion prevails, and which I am anxious to establish.

The following, then, would appear to be acknowledged and regarded by all the authorities, from Lænnec downwards, as the ratio which subsists between the pulse and the sounds of the heart, both in adults and in new born children:

Every arterial pulse at the wrist has synchronous with it an impulse in the cardiac region, and every impulse is accompanied by two sounds: first, by a loud, and more prolonged sound; and, secondly, by a feeble and more abrupt sound.—Pulse and impulse are recognized by the touch; the sounds, by the sense of hearing. Many people, indeed, find some difficulty in detecting the second sound, even in the adult state, but these are dismissed with a scornful notice by such distinguished stethoscopists as the late writer on fœtal auscultation in this journal. We may assume it, then, as being generally conceded that in the adult state the pulse and impulse range from 70 to 80, and the cardiac sounds from 70 to 80 double sounds, or from 140 to 160 single sounds. At birth, the highest authorities agree that the pulse is about 140, and consequently the cardiac sounds must be about 280; that is to say, 140 strong, and 140 feeble sounds. These are generally denominated the "tic-tac" sounds by the authorities; the "tic" meaning the strong, and the "tac" the feeble sounds. In utero no one pretends that generally either the one or the other is less frequent than at birth; and consequently we may venture to assume, as admitted, that at this period the "tic-tac" sounds, taken together, should range from 260 to 280 per minute.*

Whenever, then, an auscultator in the abdominal regions detects regularly such a low number of single sounds as 160, there can be no question that these are to be referred not to the fœtal, but to the maternal heart; at all events, most certainly not to the former. Whether, indeed, the fact be absolutely and unquestionably as stated by the celebrated auscultator in the far North, we shall not pretend to affirm or deny, but this point settled, and the inference is irresistible that these 160 single sounds cannot have proceeded from the fœtal heart. No, no; this is the standard of the adult, and not of the fœtal heart. And, by-the-way, it may be that this is the

---

*The writer referred to above, (*Medical Times and Gazette*, November 5th) makes the singular averment that "Dr. Adams is the first who affirmed that from 130 to 160 beats could be distinguished." Now this I do not affirm, but I do say that the authorities, from the first writer on it perhaps in this country (see Cyclop. of Med., p. 242) down to the latest authority, Dr. Ferguson, all speak of the fœtal sounds being double, and of excessive frequency; and the latter mentions "the tic-tac sound repeated 140 times per minute." Surely 140 brace of grouse means 280 birds all the world over! When this writer talks of 60 to 80 maternal pulsations in the minute, surely he who contends so strongly for the distinctness of the second sound of the heart, does not mean to say that this is the amount of the adult sounds. He does not mean surely that there are 30 loud and 30 feeble sounds, but 60 loud and 60 feeble ones. Altogether, however, he evidently confounds beats with sounds. But sounds are double the number of beats.

true explanation of the mistake committed by the learned auscultator in the case of spurious pregnancy related by Dr. Simpson, who may have fallen into the same mistake as our Northern auscultator in confounding the average standard with that of the fœtal heart. We think, then, the question is, in so far, set at rest, that granted 160 single sounds can be regularly detected in auscultation of the pelvic region, no one has a right to infer therefrom the existence of a living fœtal heart in that quarter.

But, it will be here asked, what if about 140 double tic-tac sounds can be detected per minute in the pelvic region, is not this an absolute and unmistakable mark that a living fœtal heart must exist within? Dr. R. Fergusson, in his Preparatory Essay to Dr. Gouch's work "On the Most Important Diseases of Women," lately published by the New Sydenham Society, answers this question in the negative. In the first place, "it is often not found;" and even when discovered, it is no certain mark of pregnancy, as the following case proves beyond controversy:

A poor girl was violated: her belly enlarged; and she was supposed to be pregnant; and, no wonder, seeing the tic-tac sound could be heard 140 times a minute. Yet it turned out to be a serous collection, as was ascertained by tapping with a trocar. It appears, therefore, that whether the allotted number of sounds be regarded as single or double, in neither case are they any certain mark of pregnancy.

We must now say a few words respecting what is usually called the *placental soufflet;* and here again we must come to an understanding regarding the general facts involved in this question.

Bellows sounds, or *bruits de soufflet*, (sounds which have not inaptly been compared to those of a locomotive just about to start on a journey,) are generally admitted to be produced more especially by the pressure of the stethoscope on enlarged vessels, whether arterial or venous, but also not unfrequently as I myself can attest, by muscular contraction, and the passage of air through an internal tube, such as the trachea in the neck, or the intestinal canal in the abdomen. Altogether these soufflets are admitted to be very fallacious guides in diagnosis; for what physician is there of experience who has not known very many cases in which vasular tumors have been thus diagnosed aneurisms, and *vice versa?* Few can have had many opportunities of seeing abdominal tumors, and not met with cases in which they were accompanied with strong *bruits de soufflet.* (See Cyclopedia of Medicine, vol. iii.

pp. 484, 485.) Dr. Meigs thus expresses the result of his experience in this matter: "As to the value of the *bruit de saufflet* in the diagnosis of pregnancy, it has been found that the earlier opinions were erroneous; and I believe there are few well-informed physicians to be now met with who give it even the smallest portion of their confidence in the unfacile discriminations that they are sometimes charged to make.—It is not to be doubted that the sound is produced by the rush of blood in vessels, and, in my opinion, sustained by a very long practice in obstetric auscultation, it depends upon the motion of the blood in the iliacs and hypogastrics. I have certainly heard the same sound after delivery as before the child was born, and I have heard it as dependent upon pressure by tumors within the abdomen. Hence I have not the least confidence in it as an object in obstetric auscultation." (*Obstetrics*, p. 240.) Dr. Fergusson, in the work quoted above, expresses himself to the same effect in the strongest terms. He has known it produced by pelvic tumors in cases of putrid fœtuses and morbid placenta; hence he holds that "it affords no diagnostic mark as to fœtal or placentry development."

After all this, what can we think of the Northen auscultator who, on the contrary, affirms that "scarcely any one at the present day entertains a doubt that in almost every case of pregnancy after the seventh month, both the pulsations of the fœtal heart and the *placental soufflet* can, with a moderate degree of care, be readily distinguished," and "that there is no possibility of a mistake being committed here;" for that "the sound of the fœtal heart can by no stretch of imagination, no flight of fancy, no exercise of faith, no intensity of impression, be confounded with any other: it is a sound *per se?*"

I am confident the writer of these sentences stands alone, at the present day, in holding the strong opinions here announced; and I shall only beg leave to add that I trust he does not reduce them to practice himself, nor encourage the youth of the profession to do so. For most assuredly if the young practitioners of the obstetric art regard it as a rule that whenever, in attending cases of protracted labor, they cannot with moderate care detect a soufflet and 160 sounds in the pelvic regions, they are quite warranted in dealing with the child as a dead body, and in applying the perforator to its head; they will soon become familiar with sights which will fill their hearts with sorrow and cover their faces with shame; sights which I forbear to describe, although I have both heard and read of such cases; but I feel grateful to Providence that albeit I am denounced as the holder of heterodox doctrines in obstetrical stethoscopy, I never was condemned to witness any such in my own practice.

With regard, then, to fœtal ascultation, I think the following points have been satisfactorily determined:—

1. That the cases of spurious pregnancy related by Dr. Simpson, in which eminent ascultators fancied they could detect the double sounds of a fœtal heart when there were none present, and the various other instances of a similar character related above, all go to prove that this process of diagnosis is not at all to be relied upon as a test of pregnancy.

2. That the leading facts of the case are so differently stated by different individuals as to put it beyond doubt that these statements must have been much modified by previous impressions and modes of faith.

3. That since soufflets are often heard in tne case of pelvic tumors, after delivery, and when the fœtus is putrid and the placenta morbid, they cannot be regarded as placentral, or as indicative of pregnancy at all.

4. That the sounds detected in the uteral region, unless double, cannot have been cardiac, nor, unless double the arterial pulse of the fœtus, can they have been connected with its heart, consequently that such an amount as from 140 to 160 single sounds cannot be referred to the fœtal heart.

5. That one of the authorities for fœtal asculation admits candidly that even this number of 140 double or tic-tac sounds is often not present in pregnancy; and, on the other hand, that it is sometimes present when there is no fœtus in utero.

6. That the fœtal heart is so surronnded by a large mass of dense maternal structures and blood-vessels, and by the solid limbs and organs of the child, that it seems next to incredible that any sound emitted by it could ever reach the ear of an auscultator.

7. That the whole system of the fœtal auscultation originated soon after the dawn of general auscultation, when men's minds were excited by the love of novelty, and warped by many erroneous impressions and mistaken modes of thinking, and has since been mainly upheld by authority.

